# Supplementary material for: High Oxytocin Receptor Expression Linked to Increased Cell Migration and Reduced Survival in Patients with Triple-Negative Breast Cancer
Source: Biomedicines. 2022 Jul 5;10(7):1595. doi: 10.3390/biomedicines10071595 (PMC9313263; doi:10.3390/biomedicines10071595)
Supplement: Supplementary file 1 [file biomedicines-10-01595-s001.zip › biomedicines-1744433-supplementary.pdf]

Supplementary Figures

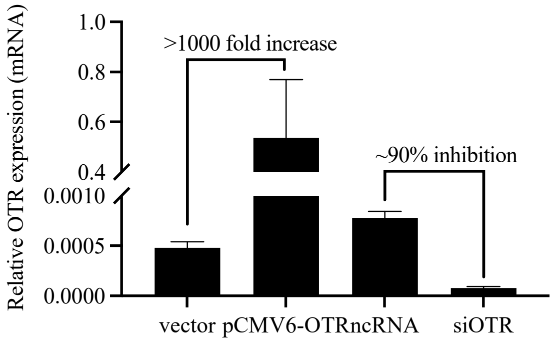

Figure S1. Efficiency of plasmid transfection for OTR overexpression and siRNA transfection for OTR knockdown. Data are means ± SD of one experiment performed in triplicate.

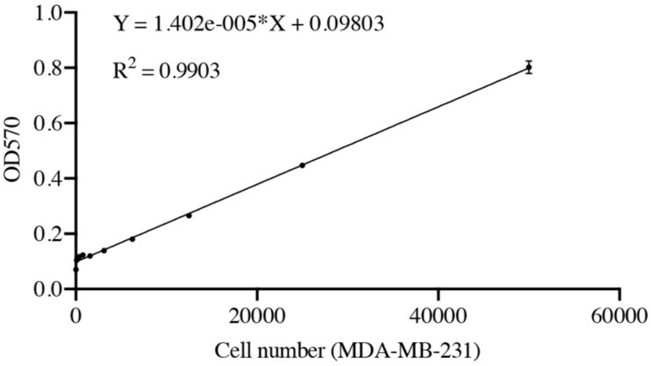

Figure S2 Standard curve of MDA-MB-231 cell number versus absorbance at 570 nm (OD570). The cells were diluted with culture medium to a series of cell densities (1:1 serial dilution from 50,000 cells/well to 97 cells/well) and seeded into a 96-well plate, medium without cells was used as negative control (no cells/well). The R^2 value of the curve was >0.99, indicating that this assay is a practical and effective approach for cell migration quantification. Data are means ± SD of one experiment performed in triplicate.

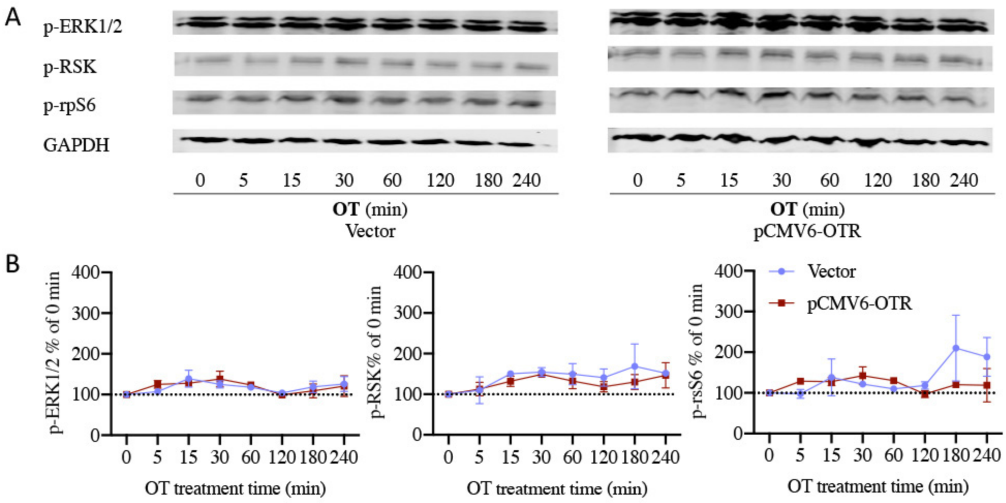

Figure S3. Representative Western blot images and quantification of phosphorylated ERK1/2, RSK and rpS6 in OT (1 μM) treated cells with or without OTR overexpression. The quantitation of ERK1/2, RSK and rpS6 phosphorylation were relative to the level of GAPDH. Vector group, MDA-MB-231 cells transfected with the vector plasmid, which were used as control cells without OTR overexpression; pCMV6-OTR, MDA-MB-231 cells transfected with the pCMV6-OTR plasmid for OTR overexpression. Data are means ± SEM of at least three independent assays. The data were analysed by two-way ANOVA followed by Tukey's multiple comparisons test.
